# Barn swallows and purple martins convert precursors to long-chain polyunsaturated fatty acids: implications for conservation of riparian- vs inland-nesting habitats

**DOI:** 10.1093/conphys/coaf068

**Published:** 2025-10-14

**Authors:** Corrine S V Génier, Matthias Pilecky, Martin J Kainz, Christopher G Guglielmo, Keith A Hobson

**Affiliations:** Department of Biology, Centre for Animals on the Move, Advanced Facility for Avian Research, University of Western Ontario, 1151 Richmond Street, London, ON, N6A 3K7, Canada; WasserCluster—Biologische Station Lunz, Inter-University Center for Aquatic Ecosystem Research, Dr. Carl-Kupelwieser Promenade 5, 3293 Lunz/See, Austria; Research Lab of Aquatic Ecosystem Research and Health, University for Continuing Education, Danube University Krems, 3500 Krems, Austria; WasserCluster—Biologische Station Lunz, Inter-University Center for Aquatic Ecosystem Research, Dr. Carl-Kupelwieser Promenade 5, 3293 Lunz/See, Austria; Research Lab of Aquatic Ecosystem Research and Health, University for Continuing Education, Danube University Krems, 3500 Krems, Austria; Department of Biology, Centre for Animals on the Move, Advanced Facility for Avian Research, University of Western Ontario, 1151 Richmond Street, London, ON, N6A 3K7, Canada; Department of Biology, Centre for Animals on the Move, Advanced Facility for Avian Research, University of Western Ontario, 1151 Richmond Street, London, ON, N6A 3K7, Canada; Environment and Climate Change Canada, Government of Canada, 11 Innovation Boulevard, Saskatoon, SK, S7N 3H5, Canada

**Keywords:** Aerial insectivore, barn swallow, compound-specific stable isotope analysis, fatty acid conversion, purple martin

## Abstract

For aerial insectivorous birds, whose populations have declined significantly in North America, long-chain polyunsaturated fatty acids (LC-PUFA) that are more abundant in aquatic-emergent insects than terrestrial insects, are important for the development, somatic growth, and health of young birds. Some bird species, however, can convert shorter chain PUFA that occur abundantly in terrestrial insects into LC-PUFA. Our study aimed to evaluate the ability of two aerial insectivore species to synthesize their own LC-PUFA. We hypothesized that terrestrially associated aerial insectivores rely on higher fatty acid conversion rates compared to those associated with wetlands and riparian habitats. We fed wild barn swallow (*Hirundo rustica*) and purple martin (*Progne subis*) nestlings ^13^C-labelled essential omega-3 (α-linolenic acid; ALA) or omega-6 (linoleic acid; LA) fatty acids to trace internal fatty acid conversion from these dietary precursors. Using compound-specific stable isotope measurements of livers, we estimated conversion efficiency to LC-PUFA. Barn swallow nestlings converted 76% of the omega-3 ALA and 46% of the omega-6 LA precursors to LC-PUFA. Purple martin nestlings converted 88% of the ALA and 44% of the LA. Both species converted five times more ALA to DHA than previously reported in tree swallows (*Tachycineta bicolor*) and may be more adapted to fluctuations in diet quality and habitat types. Our research highlights the variability in conversion efficiency within the guild of aerial insectivores and the need to better understand which species may be less resilient to sudden changes in nutritional landscapes.

## Introduction

Populations of avian aerial insectivores have declined considerably for decades in North America (Smith *et al*., 2015; Sauer *et al*., 2017; Rosenberg *et al*., 2019). However, these declines are not uniform and vary among species and regions (Michel *et al*., 2016, 2021). Several factors, including habitat loss, climate change, contaminants and insect prey declines, have been implicated (Spiller and Dettmers, 2019). Providing aerial insectivores with the necessary energy and nutrients, insect prey across North America have shown both declining (Cameron *et al*., 2011; Wepprich *et al*., 2019; Stepanian *et al*., 2020; Dalton *et al*., 2023; Rumschlag *et al*., 2023; see Hallmann *et al*., 2017; Sánchez-Bayo and Wyckhuys, 2019) and increasing population trends (Crossley *et al*., 2020; van Klink *et al*., 2020; Karatayev *et al*., 2022). Insect abundance *per se*, though influential (Møller, 2019; Shipley *et al*., 2020; Garrett *et al*., 2022), is likely not the only limiting factor for aerial insectivorous birds in North America (Imlay *et al*., 2017; McClenaghan *et al*., 2019a). Instead, the nutritional quality of nestling diets may be a significant factor (Twining *et al*., 2016; Spiller and Dettmers, 2019; Berzins *et al*., 2021). For example, Twining *et al*. (2016) found that diets rich in omega-3 long-chain (LC) polyunsaturated fatty acids (PUFA), resulted in faster somatic growth and better health of tree swallow (*Tachycineta bicolor*) nestlings compared to those with higher food quantity. Together, declines in high-quality prey may have a compounding effect with physiological consequences and cascading effects on aerial insectivore populations.

Prey quality is dependent on the primary production that initiates the nutrient pools. Primary producers synthesize omega-3 and omega-6 LC-PUFA *de novo* in quantities largely impacted by environmental conditions such as temperature (Hixson and Arts, 2016; Keva *et al*., 2021; Calderini *et al*., 2023b). Primary consumers, such as insects feeding on these primary producers, then become vectors of dietary nutrient transfer to insectivores (Dreyer *et al*., 2015; Martin-Creuzburg *et al*., 2017; Gladyshev *et al*., 2019; Mathieu-Resuge *et al*., 2021; Fehlinger *et al*., 2023). By consuming algae, aquatic-emergent insects are rich in LC-PUFA in contrast to terrestrial insects (Hixson *et al*., 2015; Twining *et al*., 2018a; Parmar *et al*., 2022; Shipley *et al*., 2022). However, nutritional landscapes are changing and valuable sources of LC-PUFA for terrestrial consumers can become limited temporally (Shipley *et al*., 2022) and spatially (Génier *et al*., 2021, 2022). Earlier insect emergences may limit LC-PUFA availability for late nesters (Shipley *et al*., 2022), and nesting away from aquatic systems also decreases availability of omega-3-rich insect prey (Génier *et al*., 2021, 2022). The omega-3 LC-PUFA eicosapentaenoic acid (EPA, 20:5n-3) and docosahexaenoic acid (DHA, 22:6n-3) are critical for neuronal and retinal development (Simopoulos, 2011; Pilecky *et al*., 2021a), and their metabolites have anti-inflammatory and pro-resolving properties. The omega-6 LC-PUFA arachidonic acid (ARA, 20:4n-6) is involved in the function of the nervous system and skeletal muscles (Shanab *et al*., 2018), and their metabolites being pro-inflammatory initiate immune responses. Studies on tree swallows have similarly shown the associated benefits of dietary omega-3 PUFA such as increased nestling growth, body condition, immunocompetence and fledging success (Twining *et al*., 2016, 2018b).

**Figure 1:**
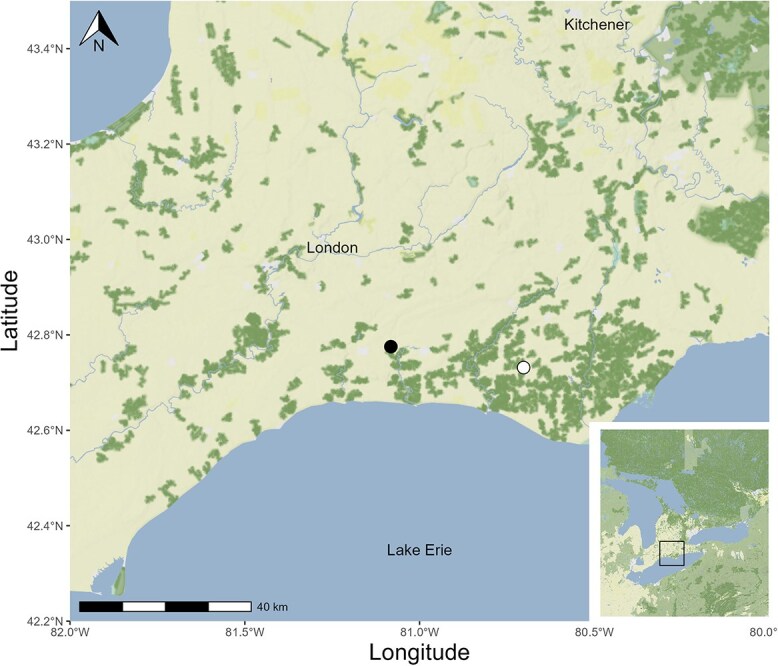
Site locations of barn swallow (*H. rustica*; black dot) and purple martin (*P. subis*; white dot) colonies, ~12 km inland from the lakeshore, used for the study in the Lake Erie region of Southern Ontario, Canada, in 2022. Each site represented a species.

In animals, LC-PUFA can also be obtained by converting shorter chain fatty acids, through the elongation and desaturation of these more readily available precursors. For example, α-linolenic acid (ALA, 18:3n-3) can be converted into EPA and DHA, and similarly, linoleic acid (LA, 18:2n-6) can be converted to ARA (Sprecher, 2000). Omega-3 and omega-6 PUFA share the same enzymes for conversion to LC-PUFA, thereby competing against each other (Simopoulos, 2011). Several terrestrial passerine species were thought to produce DHA through conversion since they had higher DHA content in pectoral muscle than aquatically associated avian species such as waterfowl (Gladyshev *et al*., 2016). Furthermore, Twining *et al*. (2018a) found nestling tree swallows were able to produce EPA and DHA from the ALA precursor, but their conversion efficiency was insufficient for their demand. Therefore, tree swallow nestlings must rely on dietary EPA and DHA for development and growth. Aerial insectivorous birds with a low fatty acid conversion efficiency and that are affected by changes in the relative abundance and phenology of aquatic versus terrestrial insects may be more vulnerable to omega-3 deficiencies than species with high conversion efficiency.

Previous studies on aerial insectivorous birds (Twining *et al*., 2016, 2018a, 2018b; Génier *et al*., 2021, 2022; Shipley *et al*., 2022), highlight the potential nutritional risks associated with reduced availability of aquatic subsidies in nestling diets. However, fatty acid conversion may help species synthesize the needed LC-PUFA from more readily available precursors. The objective of this study was to evaluate how efficiently barn swallows (*Hirundo rustica*) and purple martins (*Progne subis*) can convert dietary short-chain ALA and LA to LC-PUFA. Using isotopically labelled ALA and LA, we calculated how much of the label was found in LC-PUFA as a measure of omega-3 and omega-6 conversion, respectively. As a more terrestrial species, we predicted that barn swallows would have a higher conversion efficiency than purple martins to compensate for their lower quality terrestrial diet.

## Materials and Methods

In the Lake Erie region of Ontario, Canada, bank swallows (*Riparia riparia*), purple martins and tree swallows nesting along the lakeshore foraged on more aquatic-emergent insects and had higher plasma EPA than those nesting further inland (Génier *et al*., 2021, 2022). Barn swallows on the other hand, had the most terrestrial diet, yet had high plasma DHA regardless of nesting location (Génier *et al*., 2022). This suggests that barn swallows can convert ALA to omega-3 LC-PUFA compared to purple martins that consume dietary LC-PUFA. Using compound-specific stable isotope analysis, we compared the conversion of omega-3 and omega-6 fatty acids in barn swallow and purple martin nestlings. We conducted our field experiment at two inland study sites in the Lake Erie region of Ontario, Canada (Fig. 1). We applied and refined the protocols developed by Twining *et al*. (2018a) to fit our study system and to ensure our results would be comparable to their tree swallow results. This experiment involved administering a single dose of either uniformly ^13^C-labelled ALA or uniformly ^13^C-labelled LA oil to nestlings via gavage and then measuring the ^13^C enrichment of LC-PUFA in the liver. All animal work was approved by the University of Western Ontario Animal Care Committee (AUP 2022-028) and the Canadian Wildlife Service (SC-OR-2022-00082).

### Experimental methods and sampling

During the 2022 breeding season (May–July), field experiments were conducted on nestling barn swallows (*n* = 7) and purple martins (*n* = 7) at sites located on private land. Each site in southern Ontario, Canada, represented a single species (Fig. 1). Nests were monitored to determine hatch day. Day 7 barn swallow and Day 10 purple martin nestlings were taken from the nests and weighed to ensure we did not select significantly smaller nestlings. We selected two to three nestlings among three nests of each species that had four to five nestlings per nest. Nestlings were marked with non-toxic nail polish on their head and orally gavaged one of three types of dietary oils: (i) a control unlabelled canola oil (Saporito Foods, Markham, CAN; *n* = 1 per species), (ii) ^13^C-labelled ALA (U-^13^C18, 98%, ALA; Cambridge Isotope Laboratories, Andover, USA; *n* = 3 per species) in canola oil (5 mg/ml) or (iii) ^13^C-labelled LA (U-^13^C18, 98%, *cis*-LA; Cambridge Isotope Laboratories, Andover, USA; *n* = 3 per species) in a canola oil mix (5 mg/ml). The LC-PUFA precursors ALA and LA were both more highly enriched in ^13^C than the natural isotopic composition of a dietary precursor. When the dietary ^13^C-precursor was converted, the resulting LC-PUFA was expected to also be more enriched in ^13^C than the unlabelled naturally occurring LC-PUFA. Using pre-loaded ball-tip gavage syringes, barn swallows were gavaged with 100 μl and purple martins were given 200 μl because they weigh about twice as much. Dosages of precursors, label amount by bird mass, were 32.20 ± 1.56 mg/kg for barn swallows and 24.69 ± 1.43 mg/kg for purple martins. After 48 h, we returned to the nest, captured and humanely euthanized the nestlings under complete isoflurane anaesthesia for tissue sampling of the liver. We chose to sample at 48 h for consistency with a previous study of tree swallows by Twining *et al*. (2018a) that measured conversion of gavaged ALA into LC-PUFA in liver and muscle. Tissue samples were stored in cryovials and quickly frozen in a liquid nitrogen Dewar flask until they were later transferred to a −80°C freezer.

### Compound-specific isotope analysis of fatty acids

Lipids were extracted and fatty acids derivatized to methyl esters (FAME) at the University of Western Ontario. Compound-specific stable isotopes of fatty acids were subsequently analyzed at WasserCluster Lunz–Biological Station GmbH, Lunz am See, Austria. We used liver tissues for analysis to provide a direct comparison with previously reported results for tree swallow nestlings (Twining *et al*., 2018a).

**Figure 2:**
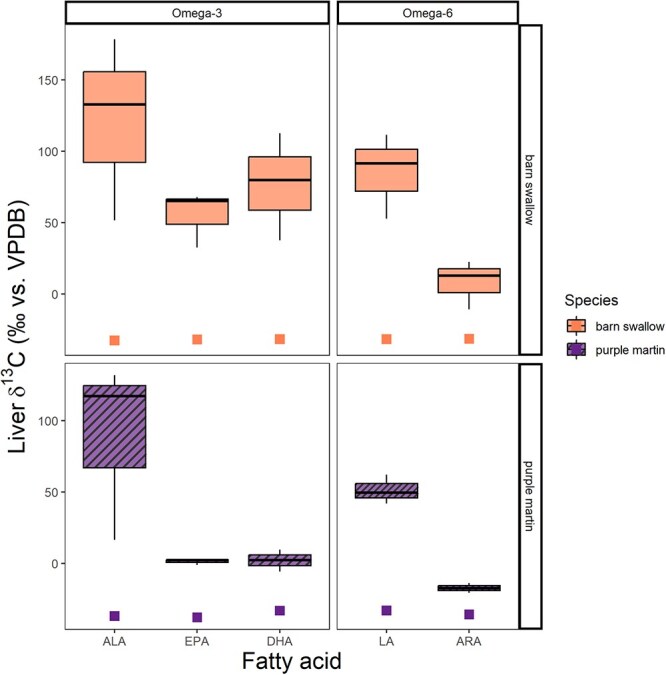
Uncorrected *δ*^13^C values (in ‰ vs VPDB) of omega-3 and omega-6 fatty acids in the liver tissues of wild barn swallow (*H. rustica*; top solid orange panel) and purple martin (*P. subis*; bottom striped purple panel) nestlings. Omega-3 fatty acids include ALA, EPA and DHA. Omega-6 fatty acids include LA and ARA. These data reflect the ^13^C-enriched fatty acids (boxplots) of nestlings fed a ^13^C-labelled omega-3 ALA or omega-6 LA, and the control nestlings (squares) fed an unlabelled oil.

**Figure 3:**
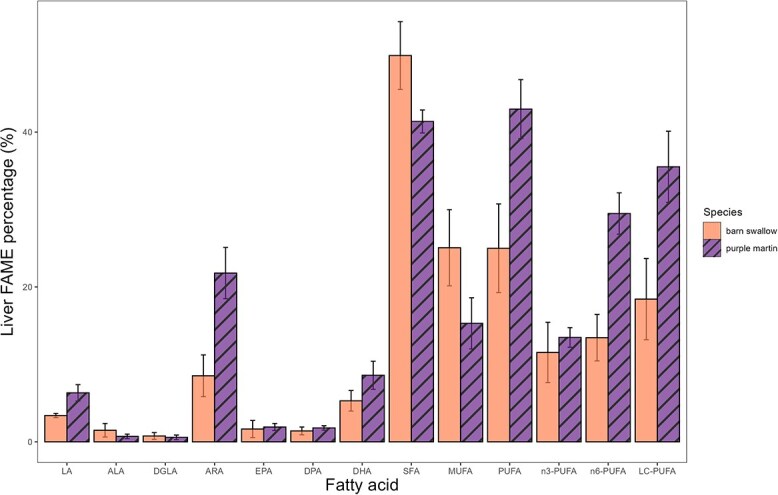
Mean % FAME ± SD in the liver tissues of wild barn swallow (*H. rustica*; solid orange) and purple martin (*P. subis*; striped purple) nestlings. Individual omega-3 and omega-6 fatty acids along with total saturated (SFA), monounsaturated (MUFA), PUFA and LC-PUFA fatty acids are depicted. Omega-3 fatty acids include ALA, EPA, DPA and DHA. Omega-6 fatty acids include LA, DGLA and ARA. Total PUFA includes all fatty acids with multiple double bonds, while n3-PUFA and n6-PUFA represent those belonging to the omega-3 and omega-6 pathways, respectively.

Sample preparation and compound-specific measurements of *δ*^13^C of fatty acids (Supplementary Material—supplementary methods) was based on a modified Bligh and Dyer (1959) extraction method and performed as described by Pilecky *et al*. (2023). Briefly, lipids were extracted using chloroform from liver samples to which we added methanol, water, butylated hydroxytoluene and an internal standard (17:0; Sigma-Aldrich, Merck Millipore Sigma, St. Louis, USA). The tubes were centrifuged, and the transferred solution was dried under N_2_ before adding methanolic hydrochloric acid and incubated in an oven. We mixed the solution with hexane and ultrapure water, and pooled the top hexane layers. FAME were separated and quantified using a gas chromatograph (Trace GC 1310, ThermoFisher) and the FA eluted were combusted to CO_2_ in a GC Isolink and analysed by an isotope ratio mass spectrometer (Delta V Advantage, ThermoFisher). Chromatograph peaks were identified by their retention time, which was set by a library of known compounds from the 37 component FAME mix standard (Supelco, Merck Millipore Sigma). Samples were standardized using a three-point calibration (USGS70/71/72). Stable carbon isotope values of each FA (Fig. 2) were expressed in the standard delta notation (*δ*) in parts per thousand (‰) relative to the international reference material Vienna Pee Dee Belemnite (^13^C:^12^C = 0.0111802) and each FA peak (Fig. 3) was expressed as a percentage of total FAME identified (% FAME).

### Data and statistical analysis

Apparent conversion efficiency calculations followed the approach of Twining *et al*. (2018a) as derived from Sheaff *et al*. (1995). First, we converted uncorrected or raw *δ*^13^C values (‰; Supplementary Material—Table S1) to atom percentages (%). The *R*_sample_ depicting the ratio ^13^C/^12^C of the sample can be derived from the standard delta notation equation (Eq. (1)), where *R*_standard_ is that of the Vienna Pee Dee Belemnite (VPDB) or 0.0112372 from Sheaff *et al*. (1995). The atom percent (AP) of each FA for each sample (Eq. (2)) was calculated by multiplying the fractionation abundance (F) for that sample by 100, where *F*_sample_ = ^13^C/(^12^C + ^13^C) = *R*/(1 + *R*).


(1) \begin{equation*} \delta^{13} \textrm{C}=[(R_{\textrm{sample}}/R_{\textrm{standard}})-1]\times 1000 \end{equation*}

(2) \begin{equation*} \textrm{AP}_{\textrm{sample}}=(R_{\textrm{sample}}/1+R_{\textrm{standard}})\times100 \end{equation*}

We then calculated the atom percent excess (APE) for each sample (Eq. (3)) as the atom percent difference between the labelled and control samples, representing birds dosed or not with an enriched FA precursor (i.e. ALA or LA).


(3) \begin{equation*} \textrm{APE}_{\textrm{sample}}=\textrm{APE}_{\textrm{labelled}}-\textrm{APE}_{\textrm{control}} \end{equation*}

We used the percentage of FAME (%; Supplementary material—Table S2) of the FA of interest *i* (e.g. EPA) rather than FA concentration as done in Twining *et al*. (2018a). We calculated total label for each FA of interest *i* in the omega-3 and omega-6 pathway by multiplying atom percent excess with percent FAME*_i_* (Eq. (4)). We recognize that factors such as body metrics and liver weight are factors to assess the amount of converted LC-PUFA. However, these variables are constants in the calculations and have no effect on the proportion of label converted or conversion efficiency, and we have chosen to remove these variables from our equation compared to Twining *et al*. (2018a). 


(4) \begin{equation*} \textrm{Total Label}_{i}=\%\ \textrm{FAME}_{i}\times\textrm{APE}_{\textrm{sample}} \end{equation*}

Conversion efficiency (CE) of each LC-PUFA product (Eq. (5)) was calculated by dividing the total labelled LC-PUFA (e.g. EPA) by the sum of all labelled FA in that pathway (e.g. omega-3 PUFA). Conversion efficiency was expressed as a percentage (%) for each conversion step in the pathway. For example, how much labelled ALA was converted into EPA is calculated:


(5) \begin{equation*} \textrm{CE}_{\textrm{ALA}\blacktriangleright\textrm{EPA}}=(\textrm{total label}_{\textrm{EPA}}/\sum\textrm{total label}_{\text{omega-3s}})\times 100 \end{equation*}

We accounted for the additional carbon of the methyl group added during methylation by correcting for the *δ*^13^C of the derivatization agent (Eq. (6); Abrajano Jr *et al*., 1994; Pilecky *et al*., 2021b, Pilecky *et al*., 2022).


(6) \begin{equation*} \delta^{13}\textrm{C}_{\textrm{FA}}=((n+1)\delta^{13}\textrm{C}_{\textrm{FAME}}-\delta^{13}\textrm{C}_{\textrm{MeOHY}})/n \end{equation*}

We used R version 4.0.5 (R Core Team, 2021), R Studio version 1.4.1717 (RStudio, 2021), and packages *ggplot2* (Wickham, 2016), *ggpattern* (FC *et al.*, 2025), *ggmap* (Kahle and Wickham, 2013), *ggspatial* (Dunnington *et al*., 2023), *cowplot* (Wilke, 2024) and *MuMIn* (Bartoń, 2020) for statistical analysis and visualization. Corrected calculations of barn swallows and purple martins were compared to those made with the method of Twining *et al*. (2018a) to determine the effect of the unlabelled methyl group. Differences between calculation approaches (Supplementary Material—Table S3) caused a significant decrease in total conversion efficiency (paired *t*-test; *df* = 11, *t* = −3.01, *P* = 0.01). However with <1% difference in conversion, we concluded that we could directly compare our results to those of Twining *et al*. (2018a) tree swallows by evaluating the conversion efficiency of each LA or ALA precursor into their respective LC-PUFA using the uncorrected calculations (see Supplementary Material—Table S3).

We compared conversion efficiencies by first taking the sum of ALA conversion to EPA, docosapentaenoic acid (DPA) and DHA as the omega-3 conversion efficiency, and similarly taking LA conversion to dihomo-γ-linolenic acid (DGLA) and ARA as the omega-6 conversion efficiency. We compared conversion efficiencies between omega-3 and omega-6 pathways and between both species in a linear regression model (LM). Using *MuMIn*, we removed the interaction between pathway and species (AICc = 107.50) but maintained the variables as fixed effects in the final model (AICc = 102.70).

## Results

**Figure 4:**
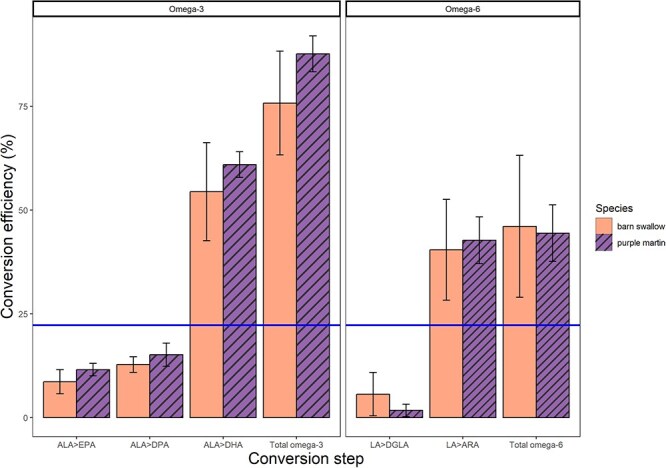
Mean conversion efficiency (% ± SD) of wild barn swallow (*H. rustica*; solid orange) and purple martin (*P. subis*; striped purple) nestlings at each step of the omega-3 and omega-6 pathways (>indicating an arrow) and the cumulative total conversion efficiency of the omega-3 and omega-6 pathway. We compared our species to previously reported omega-3 (ALA to EPA + DHA) conversion efficiency (blue solid line) of tree swallow (*T. bicolor*) nestlings (Twining *et al*., 2018a). Omega-3 fatty acids include ALA, EPA, DPA and DHA. Omega-6 fatty acids include LA, DGLA and ARA.

All nestling barn swallows and purple martins incorporated the isotopic label from the gavaged FA into liver tissues, represented by ALA and LA enriched in ^13^C (Fig. 2). Nestlings also converted these precursors into LC-PUFA (Figs 2 and 4), demonstrated by ARA, EPA and DHA enriched in ^13^C (Fig. 2). Purple martins, on average, had more LA, ARA and DHA than barn swallows (Fig. 3). The largest factors influencing conversion efficiency calculations are the stable isotope values (Fig. 2) and the proportion of fatty acids (Fig. 3). We found the addition of an unlabelled carbon during methylation had decreased conversion efficiency by <1%.

Purple martins and barn swallows converted a small proportion of ALA into EPA; 11.54 ± 1.52% and 8.61 ± 2.90%, respectively. Most importantly, both martin and swallow nestlings converted more than half of the ALA into DHA at an efficiency of 60.94 ± 3.08% and 54.40 ± 11.81%, respectively. Martins and swallows similarly converted LA into ARA by 42.70 ± 5.64% and 40.42 ± 12.14%, respectively. To reflect the conversion for each pathway, we compared total conversion efficiencies between omega-3 and omega-6 pathways and between species (Fig. 4). The total omega-3 conversion of martins (87.62 ± 4.32%) was similar to that of swallow (75.77 ± 12.52%) nestlings. Total omega-6 conversion of martins (44.42 ± 6.82%) was also similar in swallow (46.05 ± 17.10%) nestlings. The final model containing only fixed effects explained 78% of the variation. Martins and swallows did not significantly differ in conversion efficiency (LM; *df* = 9, *t* = 0.78, *P* = 0.46; Fig. 4). Total omega-3 conversion was significantly higher than omega-6 fatty acid conversion (LM; *df* = 9, *t* = −5.55, *P* < 0.01; Fig. 4).

## Discussion

Despite their differential association with riparian versus inland habitats, purple martin and barn swallow nestlings did not differ in their fatty acid conversion efficiency, contrary to our expectations. In both species, omega-3 and omega-6 conversion exceeded 75 and 40%, respectively. Most importantly, conversion of ALA to DHA was five times more than reported for tree swallows (Twining *et al*., 2018a). Although omega-3 conversion to DHA was higher than omega-6 conversion to ARA in both species, omega-6 conversion was still four times higher than the omega-3 conversion of tree swallows. Furthermore, we found that the addition of an unlabelled carbon had little influence on our calculated conversion efficiency, although this effect might be more significant in studies using natural abundances and should be considered. Overall, this study shows that purple martin and barn swallow nestlings possess efficient mechanisms to convert most of their dietary omega-3 and omega-6 precursors into LC-PUFA.

Tree swallows forage on a mix of terrestrial and aquatic-emergent insect species (McCarty and Winkler, 1999; Bumelis *et al*., 2021). However, lakeshore nestlings fed more aquatic-emergent insects acquire more omega-3 EPA than inland nestlings (Génier *et al*., 2022). In New York, tree swallow nestlings with low fatty acid conversion efficiency (Twining *et al*., 2018a) were likely reliant on the aquatic-emergent insects from adjacent waterbodies for their omega-3 PUFA. In our current study, barn swallow and purple martin colonies were further inland with surrounding agriculture. In our study area, inland and lakeshore purple martin nestlings acquire varying amounts of ARA, ALA and EPA influenced by colony proximity to Lake Erie (Génier *et al*., 2022). Purple martin nestling diet also changes with age from small soft-bodied insects to larger chitinous insects (Dunoyer *et al*., 2024). However, dragonfly carcasses were found below the inland martin colonies, suggesting older martin nestlings were provisioned with larger dragonflies. Despite being a source of LC-PUFA, predatory dragonfly fatty acid profiles can differ based on flying and perching hunting strategies (Chari *et al*., 2018) and undoubtedly influenced by their own insect prey. With such diverse nutritional landscapes and diets, purple martins would benefit from omega-6 and omega-3 PUFA conversion to compensate for the variability in diet quality or support the rapid growth of this larger aerial insectivore species. Barn swallows are generalists, but forage mainly on larger dipteran prey for their growing young (McClenaghan *et al*., 2019b; Bumelis *et al*., 2021; Kusack *et al*., 2022). In our study area, terrestrial prey, known to be low in LC-PUFA, contributed more to barn swallow diet regardless of proximity to Lake Erie (Génier *et al*., 2022). Hence, barn swallows likely use omega-3 and omega-6 PUFA conversion to compensate for a LC-PUFA deficient diet compared to more riparian foraging swallow species.

Aquatic-emergent insects are rich in omega-3 LC-PUFA, and birds would need to increase terrestrial insect biomass consumption by several fold to acquire the same amount and quality of omega-3 LC-PUFA (Shipley *et al*., 2022). Thus, even a small pulse of aquatic-emergent insects entering nestling diets can provide additional growth and health benefits (e.g. body condition and immunocompetence). However, phenological mismatching of mid- to late-nesting tree swallows and purple martins with their aquatic-emergent prey can limit access to LC-PUFA (Shipley *et al*., 2022). Furthermore, rising temperatures are correlated with a decrease in omega-3 LC-PUFA production of phytoplankton, a main source of omega-3 LC-PUFA contribution to the food chain (Hixson and Arts, 2016). Warming and eutrophication often lead to a shift in lake phytoplankton communities to green algae and cyanobacteria that produce no LC-PUFA and consequently reduce their content in aquatic insects (Keva *et al*., 2021). The production in cold and oligotrophic lakes is also limited, and consequently the greatest rates of synthesis of LC-PUFA are found in mesotrophic lakes (Calderini *et al*., 2023a, 2023b). The FA profiles of aquatic and terrestrial insects also differ during spring and summer months when many birds are breeding and raising young (Parmar *et al*., 2022; Pilecky *et al*., 2024). In the spring and early summer aquatic and terrestrial insect fatty acid profiles are the most dissimilar, where aquatic insects have a significantly larger proportion of EPA than terrestrial insects (Parmar *et al*., 2022). Rising temperatures and eutrophication are increasingly concerning for species dependent on dietary omega-3 LC-PUFA, such as tree swallows, whose conversion efficiency is low (Twining *et al*., 2018a), and possibly it is this limitation that causes nestling body condition and fledging success to correlate with insect abundance (Garrett *et al*., 2022) and composition (Twining *et al*., 2016, 2018b). On the other hand, barn swallows, having a higher omega-3 conversion efficiency, may be more resilient to fluctuations in the composition of local insect communities, and consequently insect availability may have a less pronounced effect on reproductive output (McClenaghan *et al*., 2019a). Our study indicates that purple martins are equally adaptable as barn swallows to the variation in the nutritional landscape.

For rapidly developing and growing nestlings, LC-PUFA are vital nutrients. Omega-3 LC-PUFA provide many physiological benefits to nestlings such as increased growth rate, improved body condition, increased immunocompetence and increased fledging success (Twining *et al*., 2016, 2018b, 2019). In Canada, nestling ring-billed gulls (*Larus delawarensis*) that were gavaged with fish oils had higher omega-3 LC-PUFA in their red blood cells and brain tissues, correlating with earlier fledging (Lamarre *et al*., 2021). It was suggested that higher brain DHA content improved cognition and problem solving, allowing nestling gulls to escape enclosures faster than gulls with lower brain DHA content (Lamarre *et al*., 2021). Although omega-6 PUFA may be beneficial, as suggested for hand-raised European starlings (*Sturnus vulgaris*) with lipid fuel stores high in LA expending less energy during flight (McWilliams *et al*., 2020), an excess of omega-6 PUFA can have negative effects such as inflammation susceptibility or abnormal sperm heads (Isaksson *et al*., 2017; Støstad *et al*., 2019).

Omega-3 and omega-6 PUFA often compete with each other in the phospholipid bilayer of cell membranes, for enzymes during conversion and during the formation of eicosanoids hormones (Simopoulos, 2011). Omega-3 LC-PUFA must be incorporated and utilized to be beneficial, but if omega-6 PUFA outcompete omega-3 PUFA the benefits will decrease and may instead have ramifications. Similarly, sharing the elongase and desaturase enzymes, omega-3 and omega-6 conversion processes are coupled yet also competitive (Simopoulos, 2011; Twining *et al*., 2021), and conversion rates may thus differ. Both purple martins and barn swallows had higher omega-3 conversion than omega-6 conversion. Differences between omega-3 and omega-6 conversion efficiency may be attributed to gene expression that influence regulation of enzymes (see Tu *et al*., 2010; Twining *et al*., 2021), availability of ALA and LA precursors (i.e. omega-6:omega-3 ratios) and substrate competition for enzymes (Tu *et al*., 2010; Simopoulos, 2011; Gibson *et al*., 2013), enzymatic protein concentration and activity (Valenzuela *et al*., 2024), or other factors. Nonetheless, the high omega-3 conversion efficiency of purple martins and barn swallows may help them mitigate dietary omega-3 deficiency better than tree swallows, which have a low conversion efficiency. However, little is known about the physiological requirements of omega-3 and omega-6 PUFA to support nestling growth and health, or how these requirements change with different life stages in wild birds compared to our knowledge in other animals (see Ishikawa *et al*., 2019; Rigaud *et al*., 2023). The physiological requirements and consequently conversion capacities are likely dynamic, and possibly associated with specific life stages. For example, a growing nestling or a breeding adult may have higher fatty acid conversion to support growth and reproduction than adults on non-breeding grounds that have lower maintenance costs and requirements.

In conclusion, aerial insectivores are facing changes in dietary omega-3 LC-PUFA availability with shifts in insect abundance, composition, phenology and quality among other significant factors such as climate change, changes in primary production and foraging habitats. The complex interactive effects of these dietary changes will likely not affect all aerial insectivore species or their nesting locations equally. Aerial insectivore studies will further benefit from a local understanding of insect populations and prey quality available to consumers. For example, incorporating spatio-temporal nutritional patterns into population dynamics will be invaluable information for the conservation of aerial insectivores. Our study shows how some species may have adaptive strategies that predispose them to deal with changes in diet quality, while others are more susceptible to omega-3-deficient diets. Continuing studies on conversion ability of various species such as the declining bank swallow will help identify the most vulnerable species and guide recovery strategies to consider the diet quality of nesting habitats. For this reason, we further emphasize the importance of wetland habitats and their aquatic-emergent insects, and the need to support our insect communities that provide LC-PUFA to terrestrial consumers (Hixson *et al*., 2015; Twining *et al*., 2018a; Génier *et al*., 2021; Parmar *et al*., 2022; Shipley *et al*., 2022).

## Supplementary Material

Web_Material_coaf068

## Data Availability

Data will be made available upon reasonable request. Original stable carbon isotope and fatty acid percentage data are available in the Supplementary Material.
