# Supplementary material for: Barn swallows and purple martins convert precursors to long-chain polyunsaturated fatty acids: implications for conservation of riparian- vs inland-nesting habitats
Source: Conserv Physiol. 2025 Oct 14;13(1):coaf068. doi: 10.1093/conphys/coaf068 (PMC12629658; doi:10.1093/conphys/coaf068)
Supplement: Web_Material_coaf068 [file web_material_coaf068.zip › Genier et al 2025_supplementary material.pdf]

Supplementary material

Supplementary methods:

Culture tubes were pre-baked in a muffle furnace at 450 °C for 2 hours followed by rinsing with chloroform. Liver samples were freeze-dried and homogenized into a powder in a 2 mL glass vial (Qorpak, Clinton, USA). We then added 2 mL of chloroform and stored the vials in a –80 °C freezer for 48 hours. Samples were thawed and transferred to the pre-baked culture tubes containing 1 mL of methanol, 0.75 mL of water, and 0.01 % butylated hydroxytoluene. We added 10 µL of 17:0 heptadecanoic acid (Sigma-Aldrich, Merck Millipore Sigma, St. Louis, USA) in chloroform (3 mg/mL), vortexed, and centrifuged (Allegra-6, Beckman Coulter, Brea, USA) the tubes at 2060 g for 5 minutes before transferring the solution to clean 2 mL glass vials. Samples were dried under N_2 while heated at 70 °C (reacti-vap III and reacti-therm III, Pierce, Rockford, USA). Once dried, we stored the vials at –80 °C until the next day. After thawing, 200 µL of 0.5 M methanolic hydrochloric acid (Supelco, Merck Millipore Sigma, Burlington, USA) was added to each vial. Vials were then incubated in a 70 °C oven (Isotemp, Fisher Scientific, Hampton, USA) for 2 hours for FAME formation. Vials were cooled before adding 800 µL of ultrapure water and 500 µL of hexane. Vials were then vortexed, and the top hexane layer was transferred to a clean gas chromatograph vial (Agilent Technologies, Santa Clara, USA) before repeating the hexane addition twice more. Once the organic phases were pooled, vials were dried under nitrogen gas and capped.

FAMEs were separated and quantified using a gas chromatograph (Trace GC 1300, Thermo Scientific, Waltham, USA) equipped with a HP-88 column (Agilent Technologies). The FAs eluted from the gas chromatograph were combusted to CO_2 in a combustion reactor (GC Isolink II, Thermo Scientific) coupled by a Conflo IV interface to an isotope ratio mass spectrometer (Delta Advantage, Thermo Scientific). Chromatograph peaks were identified by referencing a library of known compounds from a standard 37 component FAME mix (Supelco, Merck Millipore Sigma). Samples were standardized using a three-point calibration (USGS70/71/72). Stable carbon isotope values of each FA were expressed in the standard delta notation (δ) in parts per thousand (‰) relative the international reference material Vienna Pee Dee Belemnite (^{13}C:^{12}C = 0.0111802) and each FA peak was expressed as a percentage of total FAMEs identified.

Table S1: Uncorrected $\delta^{13}C$ values (‰ vs. VPDB) of omega-3 and omega-6 fatty acids in the liver tissues of wild barn swallow (*Hirundo rustica*) and purple martin (*Progne subis*) nestlings fed a labelled ALA (A) or LA (L) oil.

**Bird ID**	**Treatment**	**LA**	**ALA**	**ARA**	**EPA**	**DHA**
MartinC	Control	–33.0	–36.9	–35.7	–37.8	–33.1
SwallowC	Control	–31.6	–32.5	–31.4	–31.8	–31.6
MartinA1	ALA	–33.0	+16.6	–30.0	+2.6	+9.6
MartinA2	ALA	–32.8	+117.2	–32.6	–1.2	+2.4
MartinA3	ALA	–29.5	+131.8	–31.4	+2.4	–5.6
SwallowA1	ALA	–28.9	+178.4	–25.2	+67.8	+79.8
SwallowA2	ALA	–28.5	+51.7	–21.6	+65.1	+112.5
SwallowA3	ALA	–25.6	+132.9	–25.6	+32.5	+37.6
MartinL1	LA	+49.5	–40.1	–20.6	–32.8	–30.5
MartinL2	LA	+62.2	–41.9	–13.7	–30.6	–29.7
MartinL3	LA	+42.0	–39.1	–17.4	–36.2	–30.3
SwallowL1	LA	+111.4	–33.4	–10.7	–29.9	–24.2
SwallowL2	LA	+52.9	–33.9	+12.8	–37.0	–28.0
SwallowL3	LA	+91.4	–36.2	+22.5	–32.5	–26.2

Table S2: Liver omega-3 and omega-6 fatty acid percentages (%) of wild barn swallow (*Hirundo rustica*) and purple martin (*Progne subis*) nestlings fed the labelled ALA (A) or LA (L) oil.

	**Martin A1**	**Martin A2**	**Martin A3**	**Swallow A1**	**Swallow A2**	**Swallow A3**
ALA 18:3n3	0.82	0.68	0.33	1.04	1.54	2.03
EPA 20:5n3	1.67	1.67	1.39	0.75	1.42	0.81
DPA 22:5n3	1.92	2.19	1.54	1.02	1.06	1.58
DHA 22:6n3	7.39	10.07	11.06	3.56	5.85	6.37
	**Martin L1**	**Martin L2**	**Martin L3**	**Swallow L1**	**Swallow L2**	**Swallow L3**
LA 18:2n6	7.29	6.10	5.64	2.91	3.29	3.69
DGLA 20:3n6	0	0.54	0.45	0	0.59	0.71
ARA 20:4n6	23.89	19.83	23.06	7.22	7.01	8.75

Table S3: Conversion efficiency (%) of omega-3 and omega-6 fatty acids in the liver tissues of wild barn swallow (*Hirundo rustica*) and purple martin (*Progne subis*) nestlings using Twining's calculations and our corrected calculations. The > represents an arrow between precursor ALA (A) or LA (L) and their long-chain polyunsaturated fatty acid.

	**Martin A1**	**Martin A2**	**Martin A3**	**Swallow A1**	**Swallow A2**	**Swallow A3**
**Twining**						
ALA>EPA	12.932	9.917	11.784	9.326	11.084	5.421
ALA>DPA	17.953	15.110	12.349	13.864	10.582	13.824
ALA>DHA	60.697	57.993	64.136	49.465	67.878	45.855
Total	91.581	83.020	88.269	72.655	89.543	65.100
**Corrected**						
ALA>EPA	12.932	9.917	11.784	9.339	11.116	5.426
ALA>DPA	17.953	15.110	12.349	13.823	10.567	13.774
ALA>DHA	60.697	57.994	64.137	49.315	67.776	45.691
Total	91.581	83.021	88.270	72.476	89.458	64.891
	**Martin L1**	**Martin L2**	**Martin L3**	**Swallow L1**	**Swallow L2**	**Swallow L3**
**Twining**						
LA>DGLA	0	2.703	2.465	0	10.339	6.549
LA>ARA	37.560	41.794	48.732	26.404	47.253	47.593
Total	37.560	44.497	51.196	26.404	57.592	54.142
**Corrected**						
LA>DGLA	0	2.703	2.465	0	10.316	6.532
LA>ARA	37.561	41.795	48.733	26.303	47.148	47.479
Total	37.561	44.798	51.197	26.303	57.464	54.011
